# Short‐term fasting attenuates lipopolysaccharide/D‐galactosamine‐induced acute liver failure through Sirt1‐autophagy signaling in mice

**DOI:** 10.1002/mco2.412

**Published:** 2023-11-15

**Authors:** Boyu Long, Hongyun Tao, Shiwen Tong, Xuefu Wang, Wenwei Yin

**Affiliations:** ^1^ Department of Infectious Diseases, Key Laboratory of Molecular Biology for Infectious Diseases (Ministry of Education), Institute for Viral Hepatitis, the Second Affiliated Hospital Chongqing Medical University Chongqing China; ^2^ Department of Infectious Diseases Chongqing Red Cross Hospital Chongqing China; ^3^ School of Pharmacy, Inflammation and Immune‐Mediated Diseases Laboratory of Anhui Province Anhui Medical University Hefei China

Dear Editor,

Liver failure represents a serious clinical condition induced by various etiologies, and it shows a high mortality. Drug‐induced liver injury has been the most frequent factor inducing acute liver failure (ALF) because of the myriad of commonly used medications, available herbs, and dietary supplements with hepatotoxic potential. For chronic liver disease patients (like viral hepatitis, cirrhosis, and nonalcoholic fatty liver disease [NAFLD]), acute‐on‐chronic liver failure (ACLF) has been identified as the main factor leading to death.^1^ Despite the great achievements attained in exploring liver failure pathogenesis over the last 10 years, there is still a long way to go.

Calorie restriction or fasting has been considered an effective non‐pharmacological intervention for fitness and longevity. Several ongoing clinical trials are using calorie restriction as a potential therapy for various health conditions such as obesity‐related NAFLD. It has been shown that calorie restriction may help reduce insulin resistance and visceral adiposity in NAFLD patients.^2^ Lipopolysaccharide (LPS) and D‐galactosamine (D‐GalN)‐induced ALF has been the extensively constructed experimental model where tumor necrosis factor‐alpha (TNF‐α)‐mediated hepatocyte apoptosis has an important effect and LPS/D‐GalN could induce liver structure disorder and massive hemorrhage in the liver.^3^ This work focused on determining the effect of 24‐hour fasting on LPS/D‐GalN‐induced liver injury.

For analyzing how short‐term fasting (STF) affected LPS/D‐GalN‐mediated liver injury, C57BL/6 mice were fed ad libitum (AL) or fasted for 24 h and refed for diverse durations (0, 2, 6, 12, 24, 48, 60, and 72 h) prior to LPS/D‐GalN treatment (Figure S1A). According to Figure S1B, STF markedly inhibited LPS/D‐GalN‐mediated increased alanine transaminase (ALT) levels in the serum of mice refed for 2, 6, 12, 24, and 48 h but not in mice without refeeding or refed for more than 60 h compared with AL‐fed mice (Figure S1B). As STF exhibited the most significant inhibition of LPS/D‐GalN–induced ALT level in 24 h‐refed mice, we therefore arbitrarily adopted this protocol for the following experiments.

Both serum ALT and aspartate transferase (AST) levels were dramatically attenuated by STF after LPS/D‐GalN exposure in 24 h‐refed mice (Figure 1A). Among the control mice (AL group), 4 of 8 mice died within 6 h following the LPS/D‐GalN challenge, whereas only one among the six mice died from among STF mice (Figure 1B). According to HE analysis, AL‐fed mice exposed to LPS/D‐GalN exhibited prominent abnormal hepatic histological features such as massive hemorrhage, disordered hepatic lobule arrangement, inflammatory cell infiltration, and hepatocyte death, some of which were characterized by cytoplasm condensation and pyknotic nuclei (Figure 1C). However, the degree of LPS/D‐GalN‐mediated hemorrhage and liver architecture destruction decreased, albeit not eliminated, within fasting mice (Figure 1C). Anti‐apoptotic proteins FLIP, XIAP, BIRC2, Bcl‐2, and A20 of the STF group were up‐regulated compared with the control AL group (Figure S2A). By contrast, the expression of cleaved caspase‐3, a major executioner of apoptosis, was effectively inhibited in the STF group (Figure S2A). Based on the TUNEL assay, hepatocytes exhibited a high apoptosis rate within LPS/D‐GalN‐treated mouse livers, while STF significantly reversed cell apoptosis (Figure S2B). Taken together, STF prevented mice against LPS/D‐GalN‐mediated lethal hepatitis, possibly through suppressing hepatocyte apoptosis.

**FIGURE 1:**
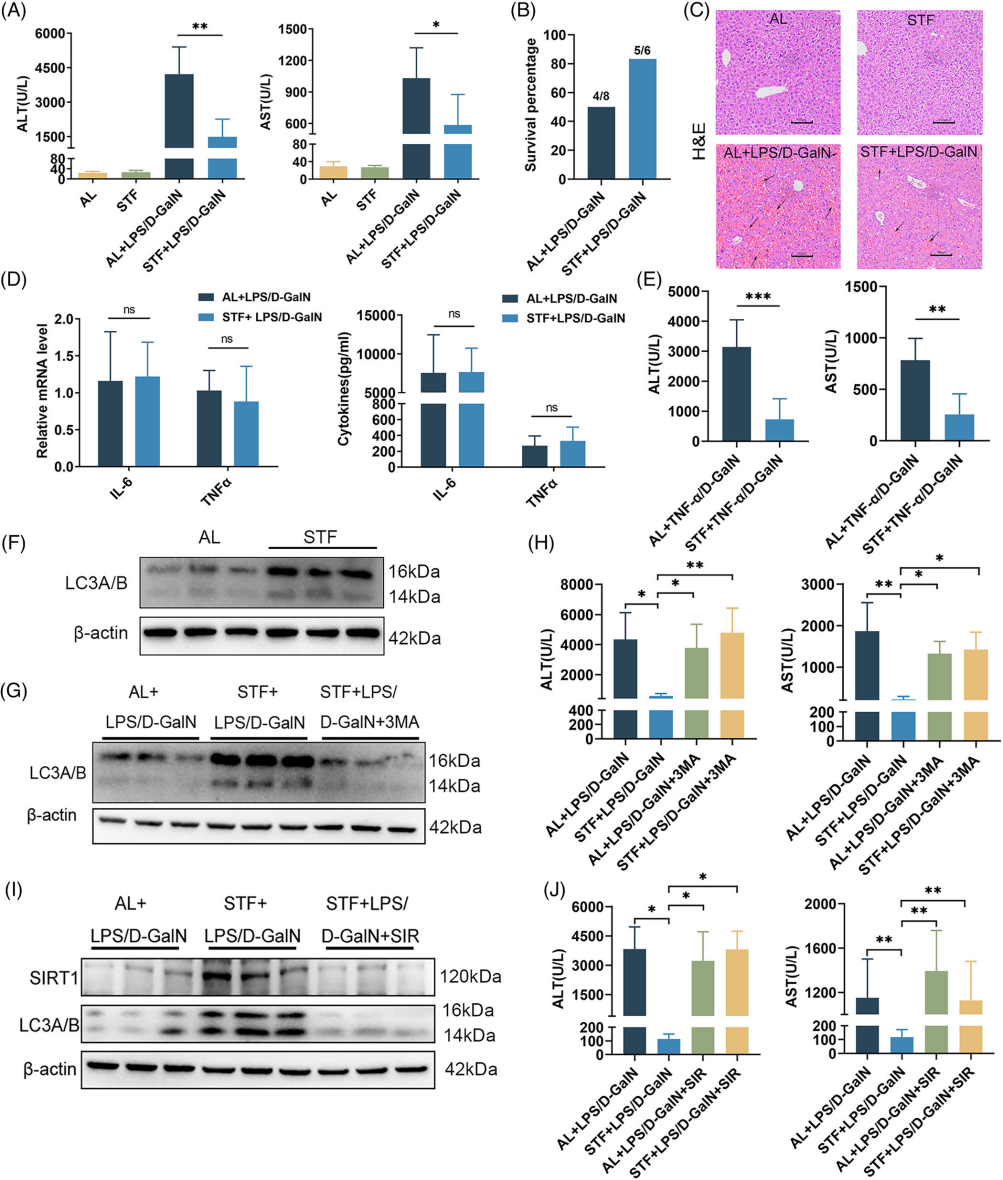
Short‐term fasting (STF) attenuates Lipopolysaccharide/D‐galactosamine (LPS/D‐GalN)‐induced acute liver failure (ALF) via the Sirt1‐autophagy pathway. (A–D) Mice were fasted for 24 h followed by a 24 h‐refeeding, subsequently, acute liver injury was induced by intraperitoneal injection of LPS/D‐GalN. (A) Serum levels of alanine transaminase (ALT) and aspartate transferase (AST), (B) the survival rates and (C) representative liver H&E staining (original magnification ×200) were shown at 6 h after LPS/D‐GalN injection (n = 5–8). The arrows indicate sinusoidal hemorrhage. (D) Intrahepatic tumor necrosis factor‐alpha (TNF‐α) and interleukin (IL)‐6 mRNA expression was measured by quantitative polymerase chain reaction (PCR), and serum levels of TNF‐α and IL‐6 were determined by enzyme‐linked immunosorbent assay (ELISA) at 2 h post‐challenge (n = 4–5). (E) The ad libitum (AL) control and STF mice were challenged with TNF‐α/D‐GalN. Serum levels of ALT and AST were analyzed at 6 h after TNF‐α/D‐GalN injection (n = 3–5). (F) LC3A/B expression in the liver from the AL control and STF mice was determined by western blot analysis. (G, H) LC3A/B expression (G) and serum levels of ALT and AST (H) at 6 h post‐LPS/D‐GalN injection following 3‐MA treatment were shown for AL control and STF mice (n = 3–5). (I, J) Sirt1 and LC3A/B expression (I) and serum levels of ALT and AST (J) at 6 h after LPS / D‐GalN injection following Sirt1 inhibition were shown for AL control and STF mice (n = 3–5). Data are presented as mean ± SD, statistical significance: ***p < 0.001, **p < 0.01, *p < 0.05.

As revealed by flow cytometry, STF made no difference to Kupffer cell proportion and Kupffer cell TLR4 expression before or after LPS/D‐GalN treatment (Figure S3B). Additionally, STF made no difference to hepatic lymphocyte (NK, NKT, CD4+T, and CD8+T cells) and neutrophil proportions, and lymphocyte activation (CD69 level) (Figure S3C,D). LPS/D‐GalN treatment significantly increased interleukin (IL)‐6 and TNF‐α mRNA expression in the liver and remarkably elevated serum TNF‐α and IL‐6 levels; however, STF did not affect their production (Figure 1D). As STF protected against LPS/D‐GalN‐mediated liver injury independent of TNF‐α inhibition, we speculate that STF probably affected hepatocyte apoptosis induced by TNF‐α. To test the hypothesis, we analyzed how STF affected TNF‐α/D‐GalN‐mediated liver failure. Likewise, STF significantly attenuated serum ALT and AST levels in TNF‐α/D‐GalN‐exposed mice (Figure 1E). Among control mice (AL group), three of seven mice died within 6 h following TNF‐α/D‐GalN treatment, whereas no mice died from among STF mice (Figure S4A). The histological examination showed severe hemorrhage and necrotic areas in the hepatic lobules of AL mice and these degenerative changes were less severe in STF mice (Figure S4B). According to the TUNEL assay, STF significantly reduced TNF‐α/D‐GalN‐induced hepatocyte apoptosis (Figure S4C). Together, these results indicate that STF prevents mice against TNF‐α/D‐GalN‐mediated fatal hepatitis and hepatocyte apoptosis.

It has been reported that calorie restriction or fasting can induce autophagy in various organs.^4^ As expected, autophagy‐related marker LC3A/B protein levels within STF mice dramatically elevated in comparison with AL‐fed mice with/without LPS/D‐GalN treatment (Figure 1F and Figure S5A,B). To determine whether autophagy regulated STF‐mediated protection from LPS/D‐GalN‐mediated liver injury, 3‐methyladenine (3‐MA) was injected to inhibit liver autophagy (Figure 1G). 3‐MA treatment did not affect serum ALT/AST concentrations within AL‐fed mice under LPS/D‐GalN treatment (Figure 1H). In contrast, 3‐MA injection almost totally reversed the reduced ALT/AST concentrations in STF mice (Figure 1H). These data were consistent with H&E staining, which showed that STF effectively reduced LPS/D‐GalN‐induced structural damage and sinusoid hemorrhage, but 3‐MA treatment abolished these effects (Figure S5C). Hepatocellular apoptosis analysis revealed that 3‐MA almost reversed the STF‐induced reduced TUNEL‐positive cell number and down‐regulated cleaved caspase‐3 expression within LPS/D‐GalN challenged mouse liver (Figure S5D,E). Taken together, the above findings demonstrate that autophagy is critical for STF‐mediated protection from LPS/D‐GalN‐mediated liver injury.

Next, we analyzed how STF regulates autophagy and alleviates ALF. Sirt1 is an important gene regulating starvation responses, which include inducing autophagic response.^5^ Sirt1 protein level in liver tissues markedly increased by STF compared to controls (Figure S6A). To determine whether or not Sirt1 mediates STF‐induced autophagy, we injected mice with sirtinol (SIR) to inhibit Sirt1 activity. Western blot and immunohistochemistry analysis showed that STF markedly increased LC3A/B levels and the STF‐induced LC3A/B expression was blocked by SIR (Figure 1I and Figure S6B), suggesting that STF‐induced autophagy is regulated by sirt1 in the liver. Sirt1 inhibition by SIR effectively abolished STF‐mediated protection from LPS/D‐GalN treatment but did not affect ALT/AST concentrations within AL‐fed mice (Figure 1J). HE staining also showed that SIR treatment abrogated the protection of STF from LPS/D‐GalN‐mediated liver architecture destruction and hemorrhage (Figure S6C). Hepatocellular apoptosis analysis revealed that SIR almost reversed the STF‐induced decrease in TUNEL‐positive cells and cleaved caspase‐3 protein level within mouse liver after the LPS/D‐GalN challenge (Figure S6D,E). So, Sirt1 inhibition by SIR treatment abrogated the protection of STF. Based on the above results, Sirt1 has an important effect during STF‐mediated protection from LPS/D‐GalN‐ALF.

To sum up, this work clearly showed a significant effect of STF in preventing severe hepatic injury resulting from LPS/D‐GalN or TNF‐α/D‐GalN. Mechanically, we demonstrated that short‐term food restriction could activate the SIRT1 signaling pathway, which regulates autophagy and attenuates the apoptosis of hepatocytes in ALF. These results suggest that Sirt1‐autophagy signaling may represent a potential therapeutic target for ALF and short‐term food restriction could be a promising dietary intervention for the prevention and management of liver diseases.

## AUTHOR CONTRIBUTIONS

B.L., H.T., S.T., and W.Y. contributed to experimental design; B.L. and H.T. were in charge of experimental implementation; B.L., H.T., S.T., and W.Y. were responsible for data analysis; B.L. and W.Y. contributed to manuscript writing. During the revision process, X.W. made great contributions to data reorganization and subsequent manuscript content modification. All authors have read and approved the final manuscript.

## CONFLICT OF INTEREST STATEMENT

The authors declare no conflict of interest.

## FUNDING INFORMATION

The present work was financially supported by the Frontier Exploration Project of Yuzhong District, Chongqing (20210130 and 2021120022), the Remarkable Innovation Clinical Research Project, The Second Affiliated Hospital of Chongqing Medical University and The First batch of key Disciplines on Public Health in Chongqing, Health Commission of Chongqing, China.

## ETHICS STATEMENT

Our animal experimental protocols gained approval from the Institutional Animal Care and Use Committee of Chongqing Medical University. The Ethics Committee of the Second Affiliated Hospital of Chongqing Medical University provided ethical approval for this work (No. 2022−94).

## Supporting information



Supporting Information

## Data Availability

Data in the present work can be obtained from the lead contact on request.
