# Supplementary material for: Short‐term fasting attenuates lipopolysaccharide/D‐galactosamine‐induced acute liver failure through Sirt1‐autophagy signaling in mice
Source: MedComm (2020). 2023 Nov 15;4(6):e412. doi: 10.1002/mco2.412 (PMC10651827; doi:10.1002/mco2.412)
Supplement: Supplementary file 1 — Supporting Information [file MCO2-4-e412-s001.docx]

**Supporting Information**

**Short-term fasting attenuates lipopolysaccharide/D-galactosamine-induced acute liver failure through Sirt1-autophagy signaling in mice**

Boyu Long^1,#^, Hongyun Tao^1,2#^, Shiwen Tong^1,*^, Xuefu Wang^3,*^, Wenwei Yin^1,*^

1. Department of Infectious Diseases, Key Laboratory of Molecular Biology for Infectious Diseases (Ministry of Education), Institute for Viral Hepatitis, the Second Affiliated Hospital, Chongqing Medical University, Chongqing, China.

2. Chongqing Red Cross Hospital, Chongqing, China.

3. School of Pharmacy, Anhui Medical University, Hefei, China.

#Boyu Long, Hongyun Tao contributed equally to this work.

*Corresponding authors

Wenwei Yin, yww@cqmu.edu.cn; Shiwen Tong, tswcqmu@cqmu.edu.cn; Xuefu Wang, wangxuefu@ahmu.edu.cn.

**Contents:** Supplementary Materials and methods (Pages 2-5)

Supplementary Figures (Pages 6-14)

**Materials and methods**

**Reagents**

LPS (*Escherichia coli*, O55:B5) and D-GalN were purchased from Sigma-Aldrich (St. Louis, MO, USA). Recombinant mouse TNF-α was purchased from Biolegend (San Diego, CA, USA). Autophagy inhibitor 3-Methyladenine (3MA) was obtained from Sigma-Aldrich (St. Louis, MO, USA). Sirt1 inhibitor sirtinol (SIR) was purchased from APExBIO (Houston, Texas, USA).

**Animal and Treatments**

Male C57BL/6 mice 8 weeks of age and weighing 20-23g were obtained from and raised at Chongqing Medical University (Chongqing, China). Mice were housed under a 12-h light-dark cycle in a barrier housing facility. The study protocol was approved by the Institutional Animal Care and Use Committee of Chongqing Medical University and the methods were carried out in accordance with the approved guidelines. Ethical approval was obtained from the Ethics Committee of the The Second Affiliated Hospital of Chongqing Medical University.

Mice were randomly divided into ad libitum (AL) groups and short-term fasting (STF) groups. In the AL group, mice had free access to food and water, while in the STF group, mice were fasted for 24h and refed for different time periods (0h, 2h, 6h, 12h, 24h, 48h, 60h and 72h) before LPS/D-GalN challenge.

Two mouse models of LPS/D-GalN and TNF-α/D-GalN were used to induce liver injury. For LPS-induced liver injury, mice were co-injected i.p., with LPS (5 μg/kg) and D-GalN (300 mg/kg). In the TNF-α/D-GalN model, TNF-α (1μg/mouse) and D-GalN(300 mg/kg) were co-injected i.p., into mice. To evaluate the potential roles of autophagy or Sir1 in STF-mediated protection against LPS/D-GalN-induced liver injury, mice were injected intraperitoneally with the autophagy inhibitor 3-methyladenine (3-MA, 30mg/kg) or the SIRT1 inhibitor sirtinol (SIR, 5mg/kg) 30 min prior to LPS/D-GalN administration.

**Transaminase Assay**

To assay for serum ALT and AST levels, we slightly anesthetized the mice with ether and gathered blood samples from the eye socket. Serum was obtained by centrifugation and stored at −20℃ until assay. ALT and AST levels were assayed by using commercially available kits (Nanjing JianCheng, Nanjing, China) in accordance with the manufacturer's recommendations.

**Measurement of Cytokine Levels**

The serum samples were kept at -20℃ until ready for cytokine measurement. The concentrations of IL-6 and TNF-α were quantified using ELISA kits commercially produced by DAKEWE (Beijing, China).

**Histopathology**

Liver samples were excised and immediately fixed in 4% paraformaldehyde solutions. The sample was embedded in paraffin wax and cut into 5 μm sections. Liver sections were attached to glass slides, dewaxed, and stained with hematoxylin and eosin (H&E) to determine morphological changes. LC3A/B (Cell Signaling Technology, Danvers, MA,USA) was detected in liver specimens using immunohistochemistry staining according to standard protocols.Apoptotic hepatocytes were detected by the terminal deoxynucleotidyl transferase-mediated dUTP nick end labeling (TUNEL) using the *in situ* Cell Death Detection Kit, POD (Roche, Mannheim, Germmany).

**Liver mononuclear cells isolation and fluorometric analysis**

The liver sample was passed through a 200-gauge stainless steel mesh and liver mononuclear cells (MNCs) were separated with 40% Percoll (GEHealthcare Pharmacia, USA). Flow cytometry analysis was performed using the following fluorochrome conjugated antibodies: APC anti-mouse CD3e (BD Biosciences # 553066), NK1.1 (BD Biosciences # 550627), F4/80 (BioLegend # 123116), Ly-6G/Ly-6C (BioLegend # 108412); PE anti-mouse CD69 (BioLegend # 104507), TLR4 (BioLegend # 145404); FITC anti-mouse CD4 (BD Biosciences # 553046), CD11b (BioLegend # 101205); Percp/cy5.5 anti-mouse CD3e (BD Biosciences # 561825), NK1.1 (BioLegend # 156256), F4/80 (BioLegend # 123128); PE/Cy7 anti-mouse CD8a (BioLegend # 100722), CD45.2 (BD Bioscience # 560696). Appropriate fluorochrome-conjugated and isotype-matched IgGs were used as negative controls. Date were recorded from the stained cells using the CytoFLEX flow cytometer (Beckman Coulter, Full-erton, CA, USA) and analyzed using FlowJo software (Tree Star, San Carlos, CA, USA).

**Western Blot**

Liver tissue was homogenized in lysis buffer for preparation of whole protein extracts. 20µg of sample protein were separated by SDS/PAGE and then transferred to 0.2μm nitrocellulose membrane by using a Criterion™ Blotter (BioRad). Nitrocellulose membranes were blocked with 2.5% BSA, followed by incubation with primary antibodies against LC3A/B (Cell Signaling Technology, Danvers, MA,USA), XIAP (Cell Signaling Technology, Danvers, MA,USA), A20 (Cell Signaling Technology, Danvers, MA,USA), SIRT1 (Origene, Rockville, USA), Flip (Affinity Biosciences, Jiangsu, China), Cleaved Caspase-3 (Affinity Biosciences, Jiangsu, China), BIRC2 (Affinity Biosciences, Jiangsu, China), BCL-2 (Abcam, Cambridge, MA, USA) and β-actin (Servicebio, Wuhan, China). ECL detection system was used to capture chemiluminescence (Bio-Rad, USA)

**Real-time PCR**

Total RNA was isolated from liver tissue by using the RNApure Total RNA Fast Exraction Kit (Bio Teke, Jiangsu, China). RNA was reverse-transcribed into cDNA by using the PrimeScript^TM^ RT reagent Kit (TaKaRa, Beijing, China). Quantitative PCR was performed using TB Green^TM^ Premix Ex Taq^TM^ Ⅱ (TaKaRa, Beijing, China).Primer sets (sense and anti-sense sequences) for the genes were as follows: GAPDH, CATCACTGCCACCCAGAAGACTG (forward) and ATGCCAGTGAGCTTCCCGTTCAG (reverse); IL-6, TACCACTTCACAAGTCGGAGGC (forward) and CTGCAAGTGCATCATCGTTGTTC (reverse); TNF-α, GGTGCCTATGTCTCAGCCTCTT (forward) and GCCATAGAACTGATGGAGGGAG (reverse); Relative changes in gene expression were calculated using the ΔΔCt method normalizing to GAPDH and relative to the control samples.

**Statistical analysis**

Dates are presented as the mean ± SD for at least three independent experiments. The results were analyzed using the Mann-Whitney U test. P-values less than 0.05 were considered as statistically significant.

**Supplementary Figures**


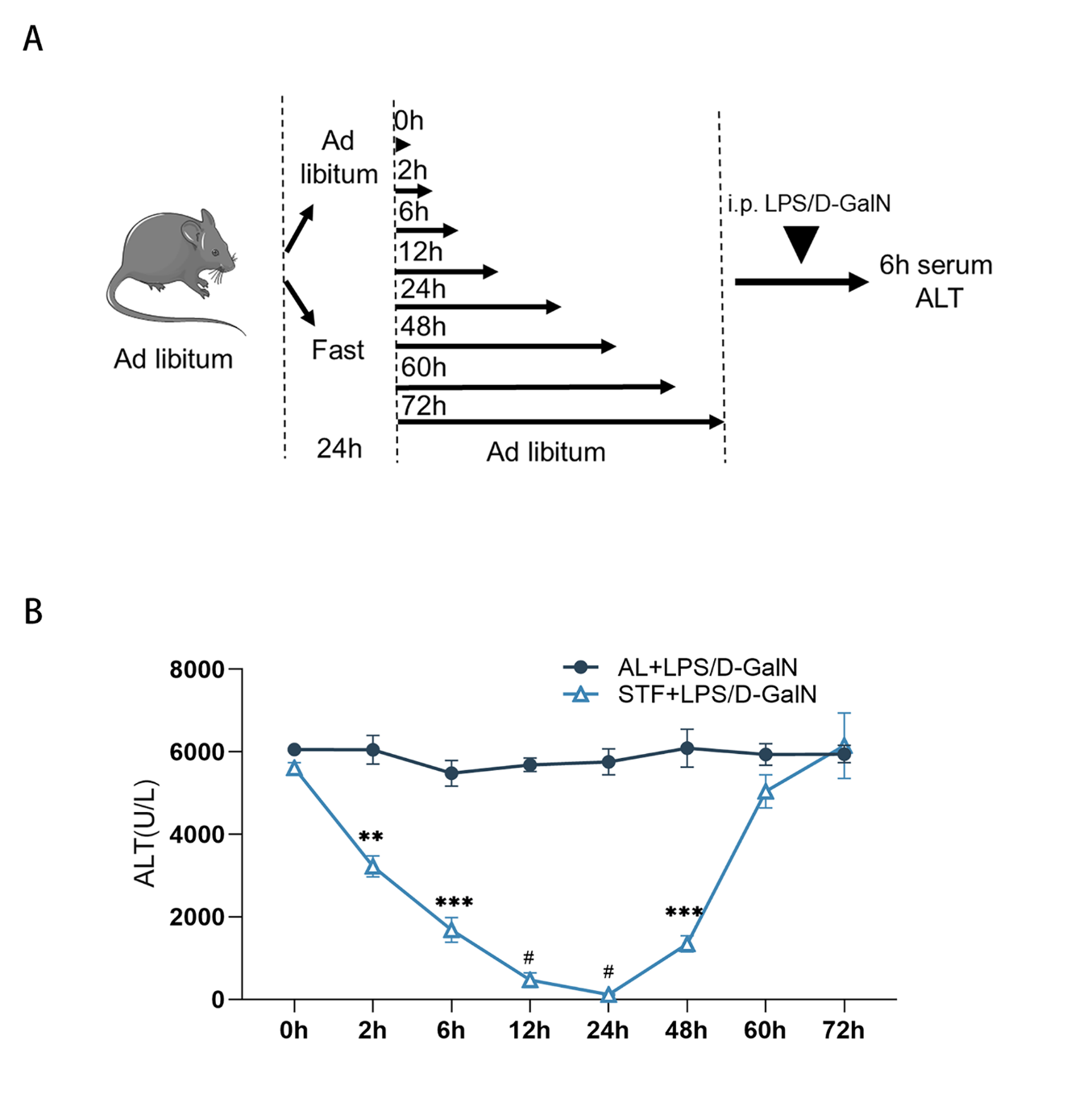


**Supplementary Figure 1. STF ameliorates LPS/D-GalN-induced acute liver injury.** (A) Schematic of the fasting/refeeding experiment. (B) Mice were fasted for 24h, refed for varying periods of time, and then subsequently intraperitoneally injected with LPS plus D-GalN. Hepatic injury was evaluated by measuring serum ALT levels 6 h after LPS/D-GalN injection (n=3-5). Data are presented as mean ± SD, statistical significance: #P < 0.0001, ***p < 0.001, **P < 0.01.

**
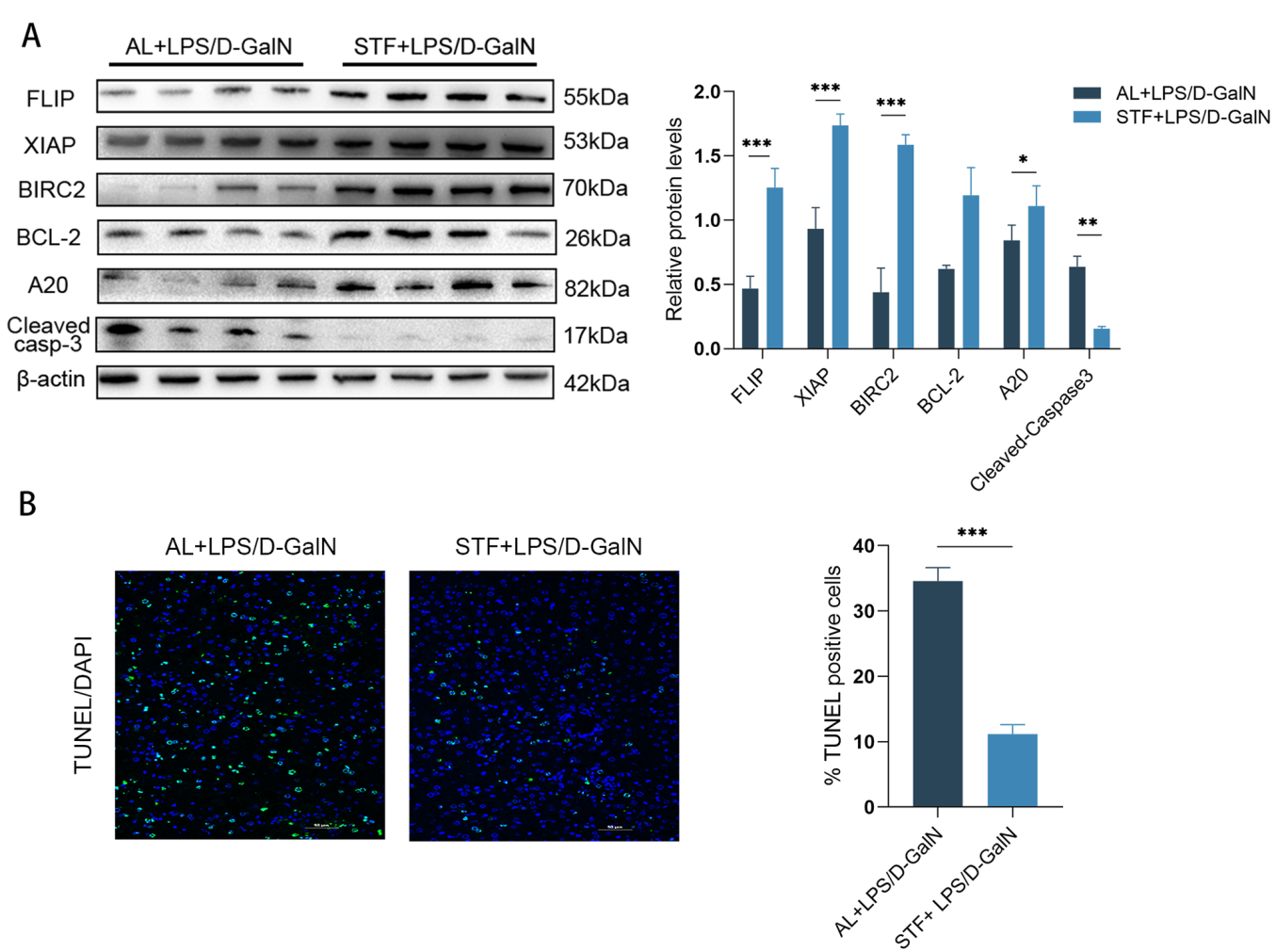
**

**Supplementary Figure 2. STF inhibits hepatocellular apoptosis in mice subjected to LPS/D-GalN.**

The AL control mice and STF mice were challenged with LPS/D-GalN. (A) Western blot analysis of the levels of FLIP, XIAP, BIRC2, BCL-2, A20, cleaved caspase-3, and β-actin. (B) Hepatocellular apoptosis was evaluated by in situ TUNEL assay (apoptotic cells are stained in green) and the number of apoptotic cells was quantified in six high-power fields (200×) and expressed as a percentage of total cells. Data are presented as mean ± SD, statistical significance: ***p < 0.001, **P < 0.01, *P < 0.05.

**
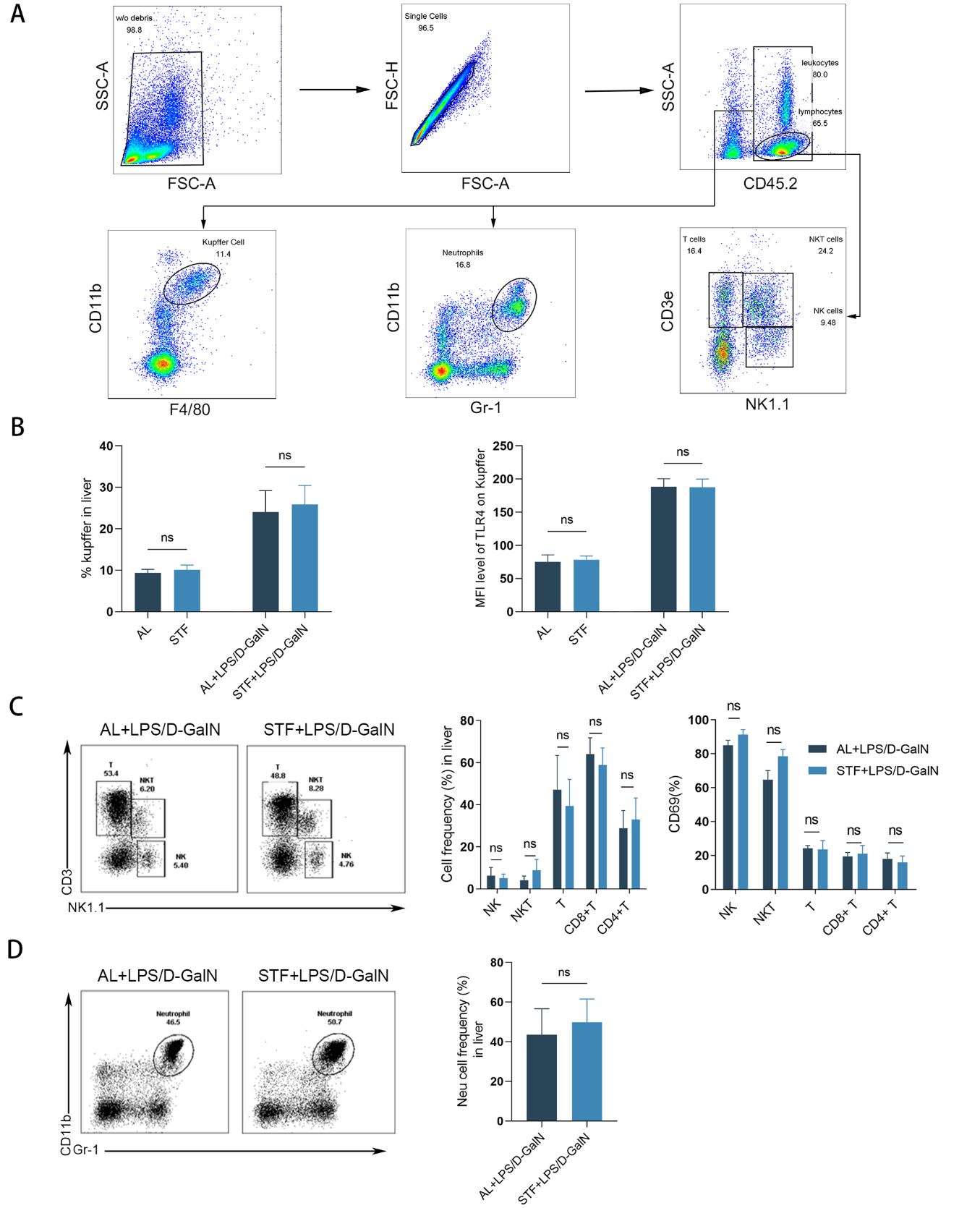
**

**Supplementary Figure 3. STF does not affect hepatic leukocyte infiltration in LPS/D-GalN mouse model.**

(A) The gating strategy for hepatic leukocyte subsets was shown. (B, C, D) Hepatic mononuclear cells (MNCs) from the AL control and STF mice were isolated, flow cytometry was applied to analyze the proportion of Kupffer cells and the MFI of TLR4 on Kupffer cells (B), the proportions of total T cells, CD4+T cells, CD8+T cells, NK cells, and NKT cells and the expression of CD69 on these cells (C), and the percentage of neutrophils (D). Data are presented as mean ± SD. n=4-5

**
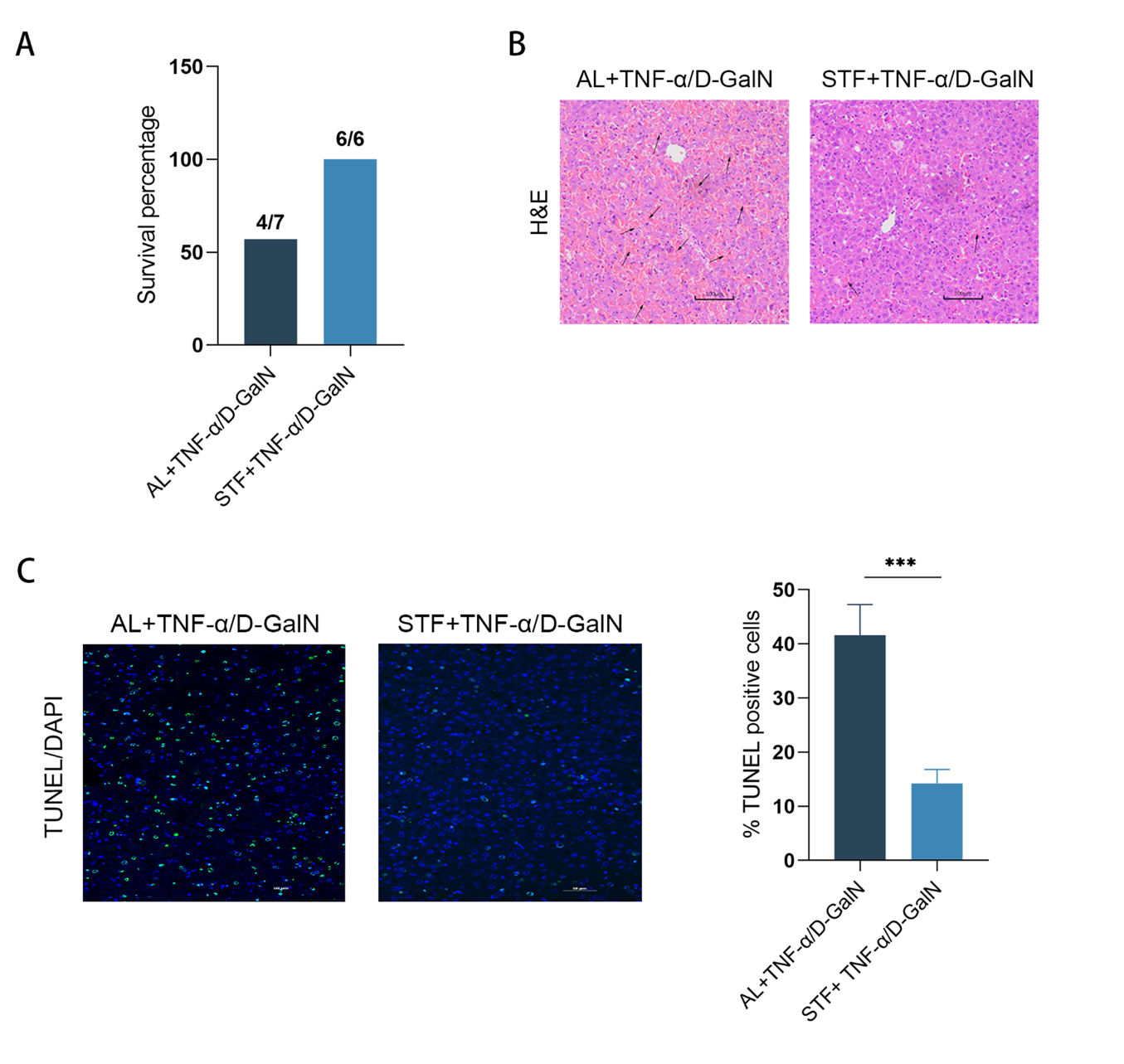
**

**Supplementary Figure 4. STF alleviates** **TNF-α/ D-GalN induced liver damage and hepatocyte apoptosis.** (A, B, C) The AL control mice and STF mice were challenged with TNF-α/D-GalN. the survival rates(A), liver H&E staining (original magnification ×200) (B), and hepatocellular apoptosis by TUNEL staining(C) were analyzed at 6h after TNF-α/ D-GalN injection (n=3-5). Data are presented as mean ± SD, statistical significance: ***p < 0.001.


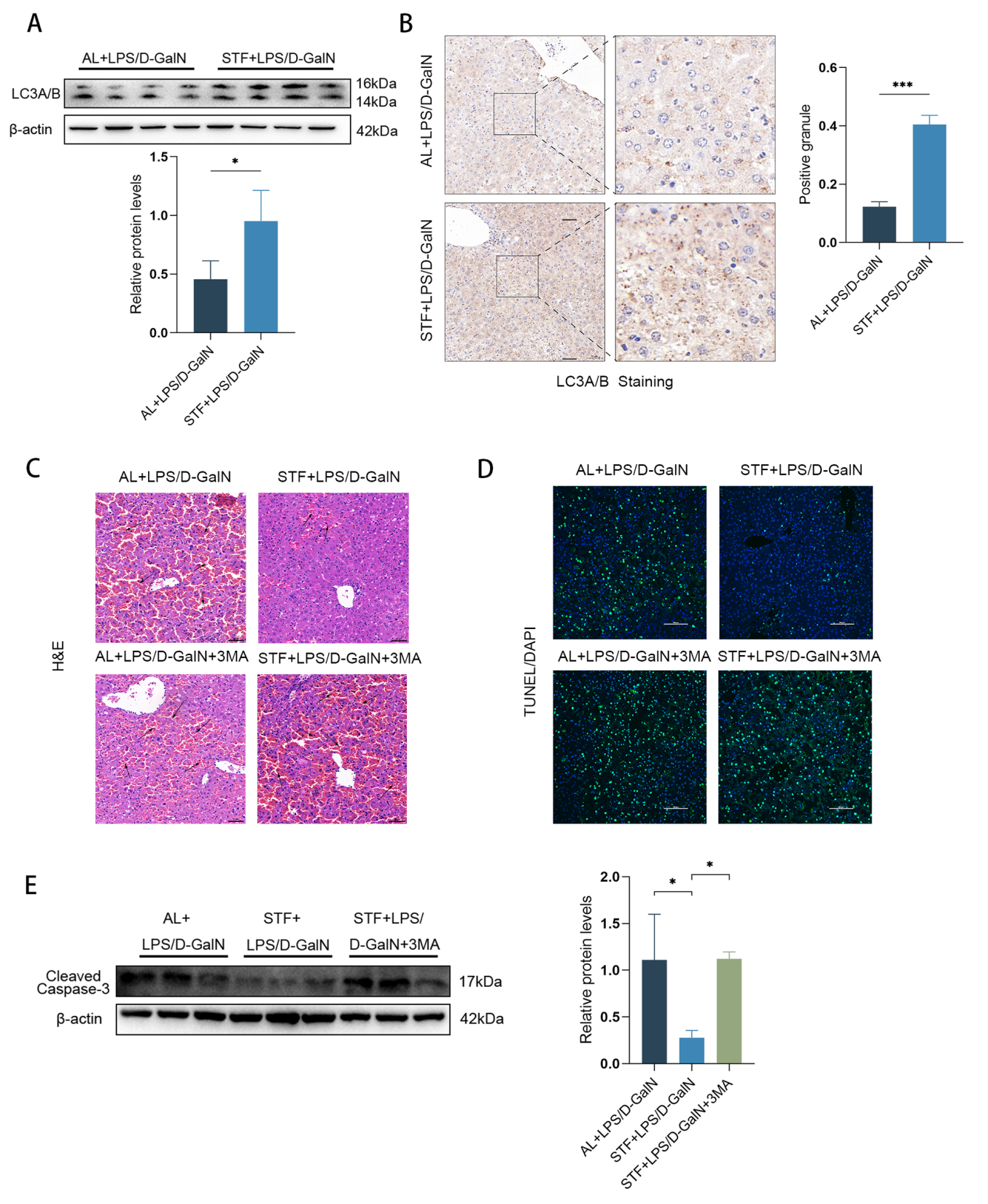


**Supplementary Figure 5. STF alleviates LPS/D-GalN induced liver injury by activating autophagy in the liver.** (A, B) The AL control mice and STF mice were challenged with LPS/D-GalN for 6h. LC3A/B expression in liver was determined by both western blot analysis (A) and immunohistochemistry (B). Positive cells were quantified in six high-power fields (200×) and expressed as a percentage of total cells. (C, D, E,) Liver H&E staining(×200) (C), hepatocellular apoptosis by TUNEL staining(×200) (D), and cleaved-Caspase3 expression(E) at 6h after LPS / D-GalN injection following 3-MA treatment are shown for the AL control mice and STF mice. n=3-5. Data are presented as mean ± SD, statistical significance: ***p < 0.001, *P < 0.05.

**
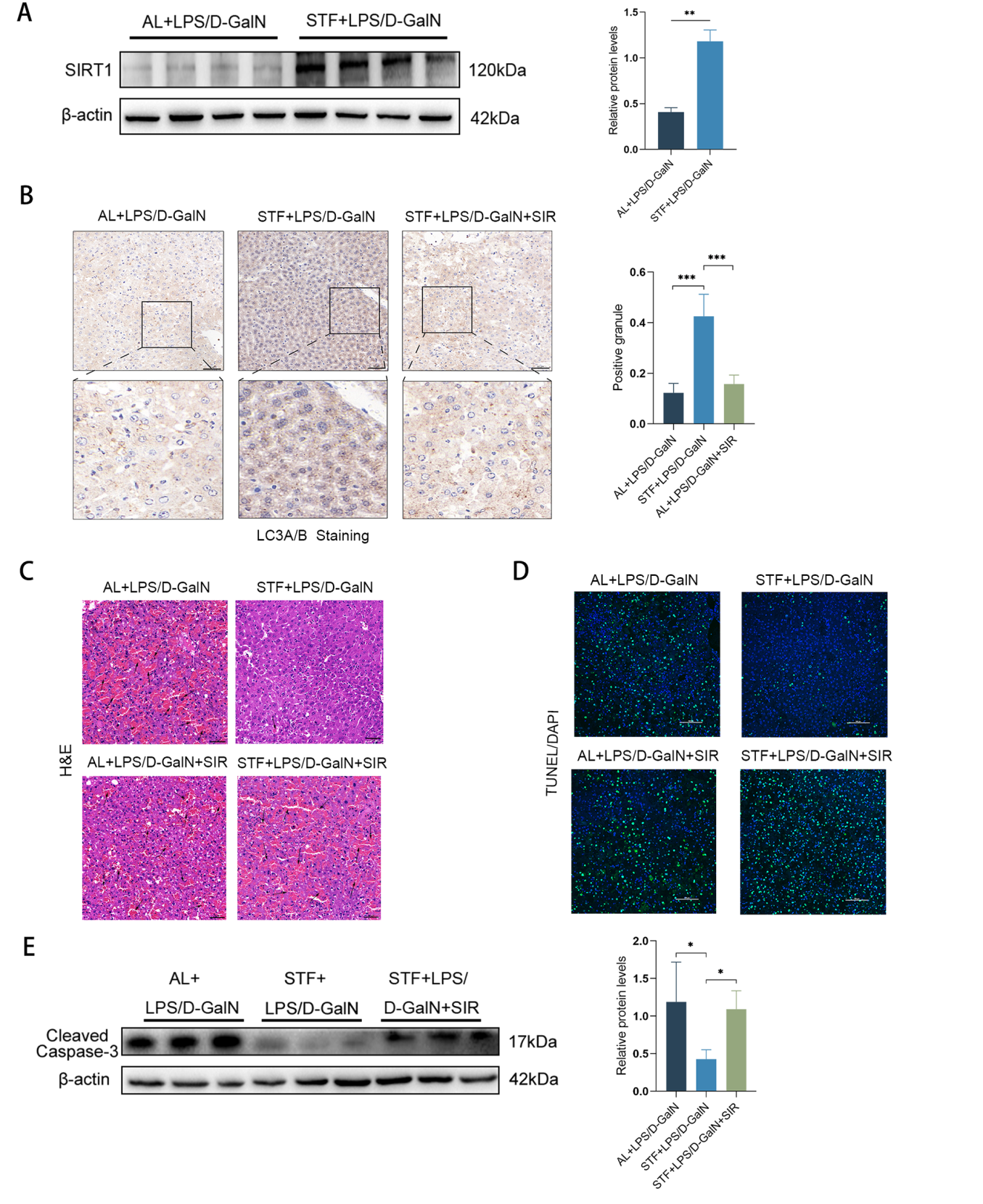
**

**Supplementary Figure 6. Sirt1 is required for STF-induced autophagy and STF-afforded protection against LPS/D-GalN-induced ALF.** (A) The AL control mice and STF mice were challenged with LPS/D-GalN for 6h. Sirt1 expression in liver was determined by western blot analysis. (B) Immunohistochemistry analysis of LC3A/B (6h post LPS/D-GalN injection) following Sirt1 inhibition. Positive cells were quantified in six high-power fields (200×) and expressed as a percentage of total cells. (C, D, E,) Liver H&E staining (×200) (C), hepatocellular apoptosis by TUNEL staining (×200) (D) and cleaved-Caspase3 expression(E) were analyzed at 6h after LPS / D-GalN injection following Sirt1 inhibition are shown for the AL control mice and STF mice. n=3-5. Data are presented as mean ± SD, statistical significance: ***p < 0.001, **P < 0.01, *P < 0.05.
